# Incidence, socio-economic inequalities and determinants of catastrophic health expenditure and impoverishment for diabetes care in South Africa: a study at two public hospitals in Tshwane

**DOI:** 10.1186/s12939-019-0977-3

**Published:** 2019-05-22

**Authors:** Chipo Mutyambizi, Milena Pavlova, Charles Hongoro, Frederik Booysen, Wim Groot

**Affiliations:** 1 Research Use and Impact Assessment, Human Sciences Research Council, HSRC Building, 134 Pretorius Street, Pretoria, 0002 South Africa; 2 Department of Health Services Research, CAPHRI, Maastricht University Medical Centre, Faculty of Health, Medicine and Life Sciences, Maastricht University, Maastricht, The Netherlands; 3 School of Economic and Business Sciences, University of the Witwatersrand, Johannesburg, South Africa

**Keywords:** Diabetes, Catastrophic health expenditure, Impoverishment, Inequality, South Africa

## Abstract

**Background:**

Direct out of pocket (OOP) payments for healthcare may cause financial hardship. For diabetic patients who require frequent visits to health centres, this is of concern as OOP payments may limit access to healthcare. This study assesses the incidence, socio-economic inequalities and determinants of catastrophic health expenditure and impoverishment amongst diabetic patients in South Africa.

**Methods:**

Data were taken from a cross-sectional survey conducted in 2017 at two public hospitals in Tshwane, South Africa (*N* = 396). Healthcare costs and transport costs related to diabetes care were classified as catastrophic if they exceeded the 10% threshold of household’s capacity to pay (WHO standard method) or if they exceeded a variable threshold of total household expenditure (Ataguba method). Erreygers concentration indices (CIs) were used to assess socio-economic inequalities. A multivariate logistic regression was applied to identify the determinants of catastrophic health expenditure and impoverishment.

**Results:**

Transport costs contributed to over 50% of total healthcare costs. The incidence of catastrophic health expenditure was 25% when measured at a 10% threshold of capacity to pay and 13% when measured at a variable threshold of total household expenditure. Depending on the method used, the incidence of impoverishment varied from 2 to 4% and the concentration index for catastrophic health expenditure varied from − 0.2299 to − 0.1026. When measured at a 10% threshold of capacity to pay factors associated with catastrophic health expenditure were being female (Odds Ratio 1.73; Standard Error 0.51), being within the 3rd (0.49; 0.20), 4th (0.31; 0.15) and 5th wealth quintile (0.30; 0.17). When measured using a variable threshold of total household expenditure factors associated with catastrophic health expenditure were not having children (3.35; 1.82) and the 4th wealth quintile (0.32; 0.21).

**Conclusion:**

Financial protection of diabetic patients in public hospitals is limited. This observation suggests that health financing interventions amongst diabetic patients should target the poor and poor women in particular. There is also a need for targeted interventions to improve access to healthcare facilities for diabetic patients and to reduce the financial impact of transport costs when seeking healthcare. This is particularly important for the achievement of universal health coverage in South Africa.

**Electronic supplementary material:**

The online version of this article (10.1186/s12939-019-0977-3) contains supplementary material, which is available to authorized users.

## Background

Since the World Health Assembly of 2005, many countries have committed to ensuring that their health systems offer financial protection from risks of catastrophic expenditure or impoverishment due to healthcare payments [1]. More recently, through target 8 of Sustainable Development Goal 3 (SGD 3), many countries have renewed their commitment to achieving universal health coverage by 2030 as a means to prevent financial hardship and to assure equitable healthcare outcomes. Access to healthcare is considered a basic human right. However, in many African countries, out-of-pocket (OOP) payments remain a hindrance to access healthcare and may result in financial hardships [2]. Globally, up to 150 million people suffer catastrophic healthcare expenditure yearly whilst 100 million are impoverished as a result of OOP healthcare payments [1]. It is reported that catastrophic health expenditure and impoverishment is high in countries in which OOP payments for healthcare are above 20% of total health expenditure (THE) [2]. The World Health Organisation (WHO) African region expenditure atlas shows that in 2012, 37 out of 47 countries had OOP health expenditure as a percentage of THE above 20% [2]. In South Africa, OOP health expenditure as a percent of THE was reported to be 7% in 2014 [2] which is lower than over 90% of countries within the European region [3].

Catastrophic health expenditure occurs when out of pocket health payments surpass a predefined threshold of a household resource such as household expenditure, resulting in a reduction of the household’s ability to spend on other essential items and may push the household into poverty [4, 5]. Previous studies on the incidence of catastrophic healthcare expenditure in South Africa – which have not focused on diabetes healthcare costs - report an incidence of catastrophic health expenditure ranging from 5 to 66% [6–13]. The differences in the estimated incidence is a result of differences in the study design, the data used, the different populations studied and the disease under analysis. It is reported that households with individuals with diabetes are at a high risk of experiencing financial difficulties [14] and more likely to experience catastrophic health expenditure [15]. Frequent visits to healthcare facilities for glucose monitoring, foot care and associated complications increase the cost of healthcare and risk being confronted with catastrophic healthcare expenditure.

The International diabetes Federation projections indicate that within the African region, diabetes health expenditure will double to 12.3 billion international dollars (ID) by 2045 [16]. In 2017 South Africa was reported to spend ID 1884 per person with diabetes [16]. This makes South Africa the country with the second largest mean healthcare expenditure on diabetes in the African region and this burden is projected to grow [16]. Despite this, there is a paucity of research on the economic impact of diabetes for patients and households within South Africa and Africa in general [17], in particular studies that estimate the incidence of catastrophic health expenditure due to diabetes.

To the best of our knowledge, only a single study has investigated the incidence of catastrophic health expenditure among diabetic patients in an African country setting. Okoronkwo et al. find an incidence of catastrophic health expenditure among diabetic patients attending a tertiary healthcare institution in Nigeria of 45% when using a catastrophic expenditure threshold of 30% [18]. Although the authors find that all socio-economic groups suffered catastrophic healthcare expenditure, the lowest socio-economic group had the highest incidence, indicating an unequal distribution of catastrophic health expenditure across socio-economic groups [18]. For diabetic patients this is very disastrous as the poor may be forced to forego other vital needs such as dietary diversity in order to attain health services. To date, in South Africa, studies that have assessed the costs associated with diabetes have done so from a health system, societal or government perspective [19–22]. No diabetes patient costing study has been conducted and no study has assessed the incidence and socio-economic inequalities of catastrophic health expenditure for diabetic patients seeking healthcare in public hospitals that provide subsidised healthcare.

Subsidised healthcare in South African public hospitals is provided according to a uniform payment fee schedule (UPFS), which groups patients into full paying patients and subsidised patients [23, 24]. Patients are classified as full paying if they are being treated within a public hospital by a private practitioner, are externally funded patients (for example funded by the road accident fund or medical scheme), or are non-South African citizens. Subsidised patients may either be fully or partially subsidised, depending on their ability to pay for healthcare services [23]. Patients are classified as fully subsidised if they provide proof of being social pensioners or formally unemployed. Partially subsidised patients pay according to their income level (commonly referred to as the sliding scale means test) [23]. Most healthcare charges are based on a grouping of services, meaning fees payable by patients include all costs such as consumables, overhead costs and salaries rather than itemised billing for each service [23]. The role of such OOP payments in healthcare financing, even in settings with modest healthcare bills, is of great concern given that this is a very regressive healthcare financing mechanism [25]. This creates the need to investigate financial protection measures in South African public hospitals.

Our study aims to (1) assess the health expenditure patterns of diabetic patients seeking healthcare in public hospitals that provide subsidised healthcare; (2) determine the incidence of poverty, catastrophic healthcare expenditure and subsequent impoverishment due to diabetes; (3) assess the socio economic inequalities in catastrophic health expenditure and impoverishment due to diabetes; and (4) explore the determinants of catastrophic health expenditure and impoverishment due to diabetes. A study of this nature is important for various reasons. The prevalence of diabetes is increasing rapidly and has a significant economic impact on individuals and households. The incidence of catastrophic health expenditure and impoverishment are important indicators of the extent of financial risk protection offered by the health system, which is particularly important within the context of universal health coverage.

## Methods

### Study setting

This study was done in Tshwane, one of the 5 districts in the province of Gauteng, which is the most populous province in South Africa. Tshwane accounts for approximately 24% of the province’s population making it the third most populous district in the province. In 2015, the district had an unemployment rate of 21.1% and a Gini coefficient of 0.64 [26]. The medical insurance coverage in the district was 30.5% in 2016 [27]. Approximately 87% of Tshwane’s employed population work in the formal sector whilst the remainder work in the informal sector [26]. The majority of the population in the district is African (78%). This too is the population group with the largest proportion of people living in poverty [26].

Healthcare is provided via public and private healthcare facilities. The district is demarcated into 7 sub-districts with public healthcare being delivered at many levels via a hierarchical referral healthcare system. Public healthcare is provided via a total of 68 clinics, 8 community health centres and 9 hospitals (district, regional and central/tertiary) in 2017/18 [27]. Each healthcare facility provides diabetes healthcare. Majority of patients accessing hospital based healthcare are those in need of a higher level of healthcare.

### Survey

A cross-sectional survey was conducted in 2017 at two hospitals in Tshwane, South Africa that operate diabetes clinics. The hospitals serve similar catchment populations and are accessible to the district’s urban population and outlying areas. The objective of the survey was to collect information on diabetes-related health issues, health behaviours, health expenditure related to diabetes care and diabetes management practices. The survey consisted of face-to-face interviews conducted with diabetic patients using a structured questionnaire. Four experienced research assistants were recruited to assist with the data collection process. The assistants were trained on the study protocol and data collection processes.

### Questionnaire

Questionnaire development was guided by the South African National Health and Nutrition Examination Survey data collection tool [28] and previous hospital based studies that estimated catastrophic health expenditure [18, 29]. The questionnaire was then adapted to the South African public hospital context. In order to ensure validity and reliability, the questionnaire was pre-tested with 8 patients at one of the hospitals and amendments made where necessary. The questionnaire required all expenditure and income data to be collected in South African Rands. The OANDA historical average exchange rate during 2017 was 13.2955 Rands per US dollar [30].

### Sampling

The study sample size was calculated using the single population proportion formula. The sample size was estimated at 385, assuming a prevalence of catastrophic health expenditure of 50%, confidence interval of 95% and absolute error of 0.05. We added 115 patients to this number to account for possibility that not all invited patients would agree to be interviewed. All patients above the age of 21 visiting the hospitals diabetes clinics during the data collection period were invited to participate whilst sitting in the waiting room before consultation. No inducement or incentive was offered for participation. Thus, a total of 503 patients were invited to take part in the survey and 405 (81%) patients agreed to be interviewed. Of those who refused to take part in the survey 62% were female and 72% were African. Of those who agreed to be interviewed, 9 were excluded from this analysis because they refused to continue with the interviews. These 9 respondents were mostly male and non-African.

### Ethical approval

Ethical approval for data collection was obtained from the Research Ethics Committee of the Human Sciences Research Council (HSRC) (ref: 14/23/11/16) and the University of Pretoria Research Ethics Committee (Protocol number 114/2017). Written consent was obtained from each participant. Clinic managers were informed of the study and permitted access to the study sites and patients. Quality checks of all interviews and validation of completed questionnaires was conducted by the data collection supervisor.

### Statistical analysis

Our study makes use of a cost of illness prevalence based approach and patient perspective to assess the OOP health expenditure incurred by diabetic patients. In this study, OOP health expenditure included payments made by diabetic patients at the hospitals. This fee does not vary for controlled versus uncontrolled diabetes and excludes OOP payments made for transportation to the health facility. However, transport costs in South Africa have previously been reported to take up a large portion of direct healthcare costs [6, 8]. Therefore, in estimating costs per hospital visit we make use of two approaches. The first uses the direct medical health costs only (approach 1) whilst the second uses both the direct medical health costs plus the direct non-medical costs of transport (approach 2). Patients were asked how much they paid for transport to the hospital and this was multiplied by two in order to estimate the costs of a return trip. In cases where patients used private vehicles, transport costs were estimated using the reported distance from patient residence to the hospital. A value for 1 km of R3.55 was used based on the price estimate published by the South African government [31].

### Definition of variables

- *Household –* A group of people living together who shared expenditures, was accepted to be a household.
- *OOP health expenditure* - Individuals were asked how much in total they paid OOP for their visit related to diabetes care. This was a fixed fee which included all services and typically included items such as consultation fees and medication
- *Total household consumption expenditure* (THE_h_) – This included expenditure made by households in order to meet their daily needs and also included expenditure on goods and services. Data were collected with specific reference to the last 30 days
- *Food expenditure* (FEH_h_)- This expenditure was measured as the amount spent on foodstuffs. Data were collected with specific reference to the last 30 days

### Measuring catastrophic health expenditure and impoverishment

The measurement of catastrophic health expenditure and impoverishment has been discussed extensively in the literature [4, 32–36]. Regardless of the method, however, a choice has to be made regarding the threshold to use in determining catastrophic health expenditure and a choice in defining the household resources used to pay for healthcare [5]. Whilst the choice of threshold is arbitrary and has typically varied between 10 and 40%, there have been two commonly used methods employed in defining household resources and measuring catastrophic health expenditure in the literature.

The first method by Wagstaff and van Doorslaer [32] defines health expenditure as catastrophic when it exceeds a certain threshold of total expenditure or household income [32, 33]. Critics of this method have argued that they underestimate the financial impact of health costs among poorer households due to the use of uniform thresholds [34, 36, 37]. The second method by the WHO (further referred to as the ‘WHO standard method’) defines health expenditure as catastrophic when it exceeds a certain threshold of capacity to pay [4, 38]. There however are some reservations with this method, which are related to how exactly subsistence expenditure is measured [32, 35, 36] and how relevant the initial estimate of the equivalence scale is [39]. More recently, some authors have argued that in order to ensure fair and ethical measures of catastrophic health expenditure, the threshold applied in measuring it should be a function of the income distribution [34, 37]. Ataguba proposes a method that uses a threshold that varies with income when estimating catastrophic health expenditure [34]. This method (further referred to as the ‘Ataguba method’) has recently been applied in measuring catastrophic health expenditure in Swaziland and Uganda, and is useful in countries with high inequalities [40, 41]. To check the robustness of our results, we employ both the ‘WHO standard method’ and the ‘Ataguba method’ in estimating catastrophic health expenditure. In both methods the incidence of catastrophic health expenditure is defined as the proportion of patients attending the diabetes clinics, whose healthcare expenditure due to diabetes is catastrophic. The steps followed in calculating catastrophic health expenditure and the extent of impoverishment are provided below, with each of the two methods being discussed in turn.

### Construction of statistical variables

- WHO standard method

The computational steps used to generate the variables used in the method by WHO [4] are shown below. A detailed description of the steps followed in constructing these variables is provided elsewhere [4].

*Step 1*: Generate the food expenditure share (FES)

$$ {FES}_h={FEH}_h/{THE}_h $$

*Step 2*: Generate the household equivalent size (HES) as follows:

$$ {HES}_h= hh\_{size}^{\beta } $$

Where hh_size is the household size, and the coefficient β is the value of an equivalence scale. Our study makes use of β = 0.56 which was estimated from a regression equation based on 59 countries [38]. A study by Koch has queried the applicability of the estimate because most of the data used to calculate it were more than 2 decades old [39]. The use of a range of scales is therefore recommended [39]. However in the case for South Africa, Koch finds that although the scale has changed over the years, the choice of scale does not really affect the average incidence of catastrophic health expenditure [39]. For the purposes of this study and consistent with recent studies [29, 42], we make use of the commonly applied household scale multiplier of 0.56

*Step 3*: The equivalent food expenditure is obtained as follows:

$$ {EFE}_h={FEH}_h/{HES}_h $$

*Step 4*: Identify the FES at the 45th and 55th percentile across the entire sample and name them FES45 and FES55.

*Step 5*: Calculate the average of food expenditures of the households that lie within FES45 and FES55 to obtain the poverty line (PL).

*Step 6*: Subsistence expenditure for each household is then calculated as follows.

$$ {SE}_h= PL\ast {HES}_h $$

*Step 7*: Generate the household’s capacity to pay (CTP_h_) which is defined as the household’s non-subsistence expenditure (SE_h_) as follows:

$$ {CTP}_h={THE}_h-{SE}_h\kern0.5em \mathrm{if}\kern0.5em {FEH}_h>={SE}_h $$

$$ {CTP}_h={THE}_h-{FEH}_h\kern0.5em \mathrm{if}\kern0.5em {FEH}_h< SE $$

*Step 8*: Health expenditure is defined as catastrophic if OOP health expenditure exceeded a certain threshold (e.g. 10%) of the household’s CTP_h_.

$$ {cata}_h=1\kern0.5em \mathrm{if}\kern0.5em {OOPHE}_h/{CTP}_h>=10\% $$

$$ {cata}_h=0\kern0.5em \mathrm{if}\kern0.5em {OOPHE}_h/{CTP}_h<10\% $$

There is a lack of consensus on the appropriate threshold to use when measuring catastrophic health expenditure. Lower thresholds are typically used in the total expenditure method and higher thresholds in the capacity to pay method [5]. Consistent with other studies the sensitivity of the analysis to various thresholds was tested [29, 43, 44] . Since the selection of threshold is a normative and somewhat arbitrary choice we present results using thresholds set at 10, 20, 30 and 40% and leave it to the reader to determine their selection.

*Step 9*: A household is defined as poor if its THE_h_ was smaller than its SE_h_ and non-poor when THE_h_ was greater than or equal to SE_h_.

$$ {poor}_h=1\kern0.5em \mathrm{if}\kern0.5em {THE}_h<{SE}_h $$

$$ {poor}_h=0\kern0.5em \mathrm{if}\kern0.5em {THE}_h>={SE}_h $$

*Step 10*: A non-poor household was considered impoverished by healthcare payments once it became poor after paying for healthcare

$$ {impov}_h=1\kern0.5em \mathrm{if}\kern0.5em {THE}_h-{OOPHE}_h<{SE}_h $$

$$ {impov}_h=0\kern0.5em \mathrm{if}\kern0.5em {THE}_h-{OOPHE}_h>{SE}_h $$

- Ataguba method

In order to check the robustness of our results and due to the limitations of the WHO standard method outlined above, we also estimated catastrophic health expenditure using the method proposed by Ataguba et al. [34]. Computational steps for the method by Ataguba et al. [34] are shown below.

*Step 1*: Estimate the rank dependent threshold Z’_cat_

$$ {Z}_{cat}^{\hbox{'}}=\gamma {\left(1-\rho \right)}^{\left(\gamma -1\right)}\ast {Z}_{cat} $$

where ρ is the household’s percentile generated when households are ordered according to income, Z_cat_ is the initial threshold (an initial threshold of 10% is used in our paper), ƴ is a parameter of aversion to inequality. Following Ataguba et al. [34], we use a value of 0.8. However, for illustrative purposes we also present results when ƴ = 1. This is the case when Z_cat_ does not change across the income distribution and is similar to applying the method by Wagstaff and van Doorslaer [32].

*Step 2*: Estimate the rank dependent overshoot which shows the extent to which health cost as a fraction of total household cost exceeds the threshold

$$ {OS}_h^{\hbox{'}}=\frac{OOPHE_h}{THE_h}-{Z}_{cat}^{\hbox{'}}\kern0.5em \mathrm{if}\kern0.5em \frac{OOPHE_h}{THE_h}>{Z}_{cat}^{\hbox{'}} $$

$$ {OS}_h^{\hbox{'}}=0\kern0.5em \mathrm{if}\kern0.5em \mathrm{otherwise} $$

*Step 3*: Estimate the rank dependent catastrophic health expenditure head count ratio which shows the proportion of households that incur catastrophic health expenditure. Where E = 1 when OS_h_ > 0 and 0 when otherwise.

$$ {HC}_h^{\hbox{'}}=\frac{1}{N}\left(\sum \limits_{h=1}^N{E}_h^{\hbox{'}}\right)={\mu}_h^{\hbox{'}} $$

- *Step 4*: Using a poverty line, estimate the pre-health payment poverty head count ratio. Our study makes use of the 2017 lower bound poverty line of R758. This is a poverty line estimate generated by Statistics South Africa which takes into account both basic food and other basic needs [45] and is the preferred threshold in policy making.

$$ {H}_{pov}^{pre}=\frac{1}{N}\left(\sum \limits_{h=1}^N{P}_h^{pre}\right)={\mu}_{p^{pre}} $$

Where ${P}_h^{pre}=1$ if adult equivalent household expenditure *THE*_*h*_ < *poverty* − *line* and ${P}_h^{pre}=0$ if otherwise.

*Step 5*: Estimate the post-health payment poverty head count ratio.

$$ {H}_{pov}^{post}=\frac{1}{N}\left(\sum \limits_{h=1}^N{P}_h^{post}\right)={\mu}_{p^{post}} $$

Where ${P}_h^{post}=1$ if *THE*_*h*_ − *OOPHE*_*h*_ < *poverty* − *line* and ${P}_h^{post}=0$ if otherwise.

*Step 6*: Estimate the impoverishing impact of OOP health expenditure, i.e. the difference between the pre-payment and post-payment indices.

$$ {PI}_{H=}{H}_{pov}^{post}-{H}_{pov}^{pre} $$

A detailed description of these steps is provided elsewhere [34]. In our study, total monthly expenditure is used as a proxy for income.

- Health seeking behaviours and time costs

Data were also collected on the time spent visiting the hospital for diabetes care and work days missed due to diabetes. Patients were asked to report in hours and minutes, how much time it took them travelling to the hospital, waiting to consult the doctor, during consultation and waiting for medication. For patients who reported being employed, the number of work days missed due to diabetes over the last 30 days, were collected using a categorical variable that took on a value of 1 when respondents took half a day, a value of 2 when respondents took 1 to 4 days, a value of 3 when respondents took 5 to 10 days and a value of 4 when respondents took more than 10 days. A continuous variable was then created by taking the mid-point estimate of each category.

The indirect costs due to productivity loss were estimated for patients who reported being employed by using the monetary value of time spent seeking care and the monetary value of days missed from work. Hourly wage rate was estimated by using respondent reported monthly income and assuming patients worked 20 days a month and 8 h a day. We then follow the method applied by Oloniniyi et al. to estimate productivity losses [46]. The hourly wage rate was multiplied by the total hours spent seeking care and time taken off work due to diabetes over the last 30 days.

### Inequalities in catastrophic health expenditure and impoverishment

Our study makes use of the concentration index (CI) to measure socio-economic inequalities in catastrophic health expenditure and impoverishment amongst the diabetes patients. The CI ranges between − 1 and + 1 and is measured as twice the covariance of the catastrophic health expenditure/impoverishment variables and the ranking of the living standards variable r all divided by the mean of the catastrophic health expenditure or impoverishment variables (*μ*):

1 $$ CI=\frac{2}{\mu}\mathit{\operatorname{cov}}\left(h,r\right) $$

Our study makes use of multiple correspondence analysis (MCA) to generate the wealth index which is our living standards variable. Although there are various methods that can be used for the construction of the asset index MCA is chosen because it is the preferred technique for categorical variables [47]. Based on items included in the questionnaire, a commonly used set of living conditions and ownership of household assets were included in constructing the wealth index. Ten household conditions and assets were considered in the analysis. The full list is as follows: housing type, water and sanitation services, ownership of a television, refrigerator, 4 plate stove, radio, cell phone, computer and car. The wealth index was later categorised into wealth quintiles. The wealth index was then applied in generating our CIs.

A negative CI means that catastrophic health expenditure or impoverishment is concentrated amongst the poor whilst a positive value means it is concentrated amongst the rich. The CI takes on a value of zero when there is no socio-economic inequality meaning catastrophic health expenditure or impoverishment variable is equally distributed across the sample. Since our variables are binary this study makes use of the Erreygers corrected CI.

2 $$ E(h)=\frac{4\mu }{b-a} CI $$

Where *μ* is the mean of the catastrophic health expenditure or impoverishment variables, CI is the concentration index, b is the maximum value of the variable (in this case 1), a is the minimum value of the variable (in this case 0). We make use of STATA’s *conidex* command [48].

### Determinants of catastrophic health expenditure and impoverishment

We used logistic regression to analyse the association between socio-demographic variables and catastrophic health expenditure and impoverishment. In assessing these associations, our selection of socio-demographic variables was guided by the literature [4, 43, 49]. The individual and household variables included in our analysis are age, gender, race, and marital status, having children, education, employment status, household size and index quintile. Age was measured in years and was included as a continuous variable. Gender was included as a binary variable taking on the values 1 – male, 2 – female. Race was also included as a binary variable with 1 – African and 2 – non-African (white, coloured, Indian/Asian). Marital status was included as follows: 1 – married/living with a partner, 2 – single. Respondents were asked if they had any children, this was included as a binary variable taking on the value of 1 when the respondent had children and 0 when the respondent did not have any children. Education was categorised as 1 – primary, 2 – secondary and 3 – tertiary education. Employment status was included as 0 – unemployed and 1 – employed. The size of the household was included as a categorical variable that took on the following values; 0–1 to 4 household members, 1–5+ household members. An outline and description of these variables is provided in an additional file (see Additional file 1).

The statistical analysis was conducted using STATA version 13. In order to allow for the skewed distributions, all household income and expenditure related data are presented as means (standard deviations) and medians (percentiles). Our study reports proportions for categorical variables.

## Results

- Descriptive statistics

The characteristics of our study sample are shown in Table 1. The mean age for our sample was 52 years. The majority of the respondents were female (61%). Our sample was predominantly African (76%). About 59% of respondents reported being single, 86% reported having children, 66% reported having secondary education, and 63% were unemployed. The majority of respondents had a household size of 1–4 persons (64%).

- Cost of illness and household expenditure

**Table 1:** Study sample characteristics

Variable	N	Mean	Std. Dev.
Age	386	51.62	14.6567
Gender			
Male	155	39%	0.4889
Female	240	61%	0.4889
Race			
African	300	76%	0.4256
Non-African	93	24%	0.4256
Marital status			
Married	162	41%	0.4929
Single	231	59%	0.4929
Children			
Yes	337	86%	0.3477
No	55	14%	0.3477
Education			
Primary	65	17%	0.3739
Secondary	255	66%	0.4753
Tertiary	68	18%	0.3807
Employment status			
Unemployed	236	63%	0.4827
Employed	137	37%	0.4827
Household size			
1–4	249	64%	0.4815
5+	142	36%	0.4815

Note: N – number of observations. Std. Dev. – Standard deviation

Table 2 demonstrates that the mean total household expenditure for our sample was R4213 (1 US$ = 13.30 Rands) (median value R2810). Household food expenditure averaged R1687 (median R1500). Subsistence expenditure averaged R1003 (median R1016) and capacity to pay averaged R3325 (median value R1730).

**Table 2:** Individual and household expenditure

Variable	N	Mean	Std. Dev.	Median	p25	p75
Household costs per month						
Total Household expenditure	380	4213	5190	2810	1600	5000
Household food expenditure	385	1687	1258	1500	900	2000
Subsistence expenditure	391	1003	303	1016	689	1151
Capacity to pay (CTP)	380	3325	5133	1730	911	3725
Direct medical cost per patient per hospital visit						
Healthcare cost	377	53	167	40	0	65
Healthcare cost (including transport)	361	132	209	96	50	150
Direct non-medical cost per patient per hospital visit						
Transport cost	376	79	119	40	20	91
Food cost during hospital visit	289	30	153	20	0	30
Time Lost per patient (hours)						
Time taken off work (30 day period)	100	11	7	16	4	16
Time taken visiting hospital	151	7	2	7	6	9
Total time lost	153	14	8	11	8	22
Indirect costs per patient						
Time taken off work (30 day period)	100	429	615	250	110	450
Time taken visiting hospital	151	277	369	161	83	323
Total time lost	153	553	786	323	113	652

Note: All monetary values are presented in South African Rands, Note: N – number of observations. Std. Dev. – Standard deviation

**Fig. 1:**
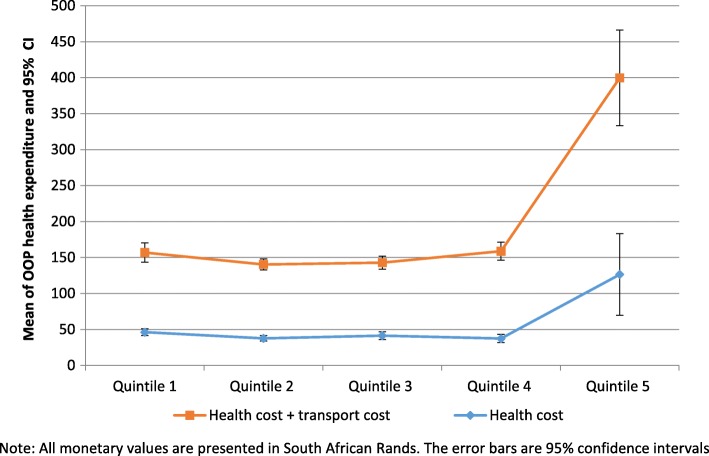
Mean of OOP health expenditure for diabetes care by socio-economic status

Our analysis suggests that the mean OOP health expenditure per out-patient visit related to diabetes care was R53 (median value R40). The mean round trip transport cost was R79 (median R40). When we combine transport costs and health costs, we find an average of R132. The OOP health expenditure was statistically significantly different across the wealth index quintiles. Households in quintile 5 pay more for health services relative to other quintiles (see Fig. 1). Other direct non-medical costs incurred by patients included expenses on food whilst waiting at the hospital. The mean food costs was R30 (median R20).

Our estimation of productivity losses is restricted to those who reported being employed and reported their income (*n* = 153). Our findings show that out of a sample of 100 patients who reported missing work over the past 30 days due to diabetes, the average time lost was 10.6 h. No respondent reported taking more than 10 days off work due to diabetes related illness. The average time for a hospital visit was 7 h. The mean estimated indirect costs for missing work over the past 30 days and for a hospital visit was R 429 and R 277 respectively. When we combined the average time for a hospital visit and the time taken off work due to diabetes, the total time lost was 14.5 h. The mean estimated indirect costs for both time spent seeking healthcare and time taken off work was R 553 (see Table 2).

- Catastrophic health expenditure and impoverishment patterns

In estimating catastrophic health expenditure and impoverishment due to healthcare costs, we employed both the WHO standard method and the Ataguba method (see methods section). As mentioned earlier, we considered catastrophic expenditure due to direct OOP payments for diabetes care (approach 1) and also catastrophic expenditure due to both direct OOP payments for diabetes care plus direct non-medical costs of transport (approach 2). The sensitivity of the analysis to different thresholds was tested and results presented in Table 3. Due to the differences in the computational steps used in the construction of statistical variables (see methods), the sample sizes differ for each analytical method (WHO method and the Ataguba method). In each method observations with missing data in variables used for the construction of catastrophic health expenditure or impoverishment were excluded from the analysis.

**Table 3:** Catastrophic health expenditure and impoverishment related to diabetes care (%)

Method/Indicator	Approach 1			Approach 2		
	N	%	Stnd.dev	N	%	Stnd.dev
WHO Standard method						
Poor	375	6%	0.2353	375	6%	0.2353
Catastrophic 10	359	9%	0.2813	343	25%	0.4357
Catastrophic 20	359	4%	0.2066	343	11%	0.3143
Catastrophic 30	359	3%	0.1726	343	8%	0.2697
Catastrophic 40	359	2%	0.1478	343	6%	0.2454
Impoverished	336	0%	0.0000	320	2%	0.1465
Ataguba method (ƴ = 0.8)						
Catastrophic head count ratio	362	4%	0.1996	346	13%	0.3400
Prepayment poverty head count	357	21%	0.4059	341	21%	0.4066
Post-payment poverty head count	357	23%	0.4212	341	25%	0.4349
Impoverished	357	2%	0.1482	341	4%	0.2053
Ataguba method (ƴ = 1)						
Catastrophic head count ratio	361	2%	0.1561	344	10%	0.2989
Prepayment poverty head count	357	21%	0.4059	341	21%	0.4066
Post-payment poverty head count	357	23%	0.4212	341	25%	0.4349
Impoverished	357	2%	0.1482	341	4%	0.2053

Note: N – Number of observations in each method, Stnd.dev – standard deviation. Approach 1 is catastrophic expenditure due to direct health costs only, approach 2 is catastrophic expenditure due to both direct medical health costs plus the direct non-medical costs of transport. Ataguba method (y = 0.8) - threshold varies with household expenditure. Ataguba method (y = 1) - constant threshold of 10%

Results from the WHO method show that approximately 6% of the study sample was poor. Catastrophic health expenditure for diabetes care was measured using four thresholds (10, 20, 30 and 40%) and decreased as we moved from the lower to higher thresholds. As expected, when we included transport costs in estimating the ratio of healthcare costs to capacity to pay, we find that catastrophic health expenditure measured at all four thresholds was much higher than when we exclude transport costs. This finding signifies the importance of transport costs. Using approach 1 (approach 2), catastrophic health expenditure for diabetes care varies from 2 to 9% (6 to 25%). Amongst those who were not poor and when we exclude transport costs, none of our study participants are impoverished due to diabetes care but when we include transport costs, 2% become impoverished.

For the Ataguba method, we make use of an initial threshold of 10% to estimate catastrophic health expenditure for diabetes care. We present results using a parameter of aversion to inequality of 0.8 (a varying threshold) and of 1 (a constant threshold of 10%). As shown in Table 3, the increase of this parameter from 0.8 to 1 decreases the head count from 4 to 2% (approach 1) and 13 to 10% (approach 2). Using the South African poverty line of R758, we find that the poverty head count increased from 21 to 23% (approach 1) and 21 to 25% (approach 2). This translates into a 2 and 4% rise in poverty using approach 1 and approach 2, respectively.

**Table 4:** Concentration indices for catastrophic health expenditure for diabetes care

Indicator	Approach 1			Approach 2		
	N	Index value	*p*-value	N	Index value	*p*-value
WHO Standard method						
Catastrophic 10%	359	−0.1246	0.0002	343	−0.2299	0.0000
Catastrophic 20%	359	−0.0575	0.0220	343	−0.1128	0.0038
Catastrophic 30%	359	−0.0247	0.2403	343	−0.0933	0.0046
Catastrophic 40%	359	−0.0143	0.4274	343	− 0.0744	0.0148
Impoverished				320	− 0.0297	0.1158
Ataguba method (ƴ = 0.8)						
Catastrophic head count ratio	362	−0.0518	0.0320	346	−0.1026	0.0148
Impoverished	341	−0.0413	0.0289	341	−0.0512	0.0460
Ataguba method (ƴ = 1)						
Catastrophic head count ratio	361	−0.0236	0.2130	344	−0.0582	0.1176
Impoverished	341	−0.0413	0.0289	341	−0.0512	0.0460

Note: N - Number of observations. Note: Approach 1 estimates catastrophic health expenditure using health costs only, approach 2 uses health costs plus transport costs. Ataguba method (y = 0.8) - threshold varies with household expenditure. Ataguba method (y = 1) - constant threshold of 10%

The proportion of diabetic patients who were poor, was highest in wealth quintile 1. The proportion of catastrophic health expenditure for diabetes care was highest in the first wealth quintile when measured using both the WHO standard method and the Ataguba method. This shows that diabetic patients who incur catastrophic health expenditure are in fact the poor. From the Ataguba method we find that higher levels of catastrophic expenditure exist within quintile 1 when measured using a variable threshold (y = 0.8) compared to when measured using a fixed threshold (y = 1). For example, in approach 2 we find an incidence of catastrophic expenditure of 20.27% within quintile 1 when measured using a variable threshold and we find an incidence of catastrophic expenditure of 16.44% within quintile 1 when measured using a fixed threshold. Using the Ataguba method, the highest increases in the poverty head count ratio is recorded for diabetic patients in quintile 1. For example, we see that OOP health expenditure for diabetic care leads to a 6.85% increase in the poverty head count ratio when using approach 1, i.e. without accounting for transportation costs. The figure for approach 2 is higher at 9.59% (see Additional file 2).

- Inequalities in catastrophic expenditure and impoverishment

Table 4 shows the concentration indices for catastrophic health expenditure and impoverishment related to diabetes care. All statistically significant concentration indices are negative, indicating that catastrophic health expenditure for diabetes care is more concentrated amongst the poor diabetic patients. In the WHO standard method, the value of the catastrophic health expenditure index decreases as we move to higher thresholds, but also declines in statistical significance. The Ataguba method (ƴ = 0.8), which uses a varying threshold, shows higher inequalities in catastrophic health expenditure when compared to the one which uses a constant threshold of 10% (ƴ = 1). However, the CI for the Ataguba method (ƴ = 1) are statistically insignificant. All the values for the concentration for impoverishment due to diabetes were negative, indicating that the proportion of impoverishment is concentrated amongst the poor. Because none of our study participants are impoverished due to diabetes care when estimated using the WHO method for approach one, we do not estimate this inequality.

- Factors associated with catastrophic health expenditure and impoverishment

This section analyses the socio-demographic factors associated with catastrophic health expenditure and impoverishment for diabetes care based on results from multivariate logistic regressions. For brevity and due to the loss of statistical power, we present results from the multivariate logistic regression analysis for four out of the 12 estimates of catastrophic healthcare expenditure, represented here by binary variables. Table 5 shows the results for the WHO standard method using a threshold set at 10% for both approach 1 (medical costs only) and approach 2 (medical and transportation costs). For the Ataguba method, we present results when catastrophic health expenditure for diabetes care is measured using approach 2 (health costs plus transport costs) when the parameter of aversion to inequality is ƴ = 0.8 and ƴ = 1. Given the low estimates of catastrophic health expenditure for Ataguba’s method when applied using approach 1, regression models were estimated only for approach 2.

**Table 5:** Factors associated with catastrophic health expenditure for diabetes care

Variable	Catastrophic health expenditure								Impoverishment			
	WHO standard method (Threshold = 10%)				Ataguba method				Ataguba method			
	Approach 1		Approach 2		Approach 2; y = 0.8		Approach 2; ƴ = 1		Approach 1		Approach 2	
	OR	SE	OR	SE	OR	SE	OR	SE	OR	SE	OR	SE
Age	0.9937	0.0184	0.9959	0.0120	1.0256	0.0158	1.0131	0.0175	1.0414	0.0468	0.9867	0.0248
Female gender	3.7193***	2.0622	1.7343*	0.5177	1.2347	0.4867	2.1125	1.0001	12.8261**	15.9804	2.9832	2.1404
Non-African	0.1620*	0.1733	1.0108	0.3688	1.0463	0.4895	1.4307	0.7103	1	–	0.2506	0.2789
Single marital status	0.6182	0.2984	0.7161	0.2097	0.8858	0.3454	0.5065	0.2210	0.0082***	0.0127	0.2991*	0.1900
No children	4.0092**	2.7129	1.3224	0.5922	3.3594**	1.8273	3.5447**	2.1681	20.380**	34.5052	1.2749	1.5671
Secondary education	1.7726	1.1278	0.8512	0.3249	1.0830	0.5281	0.8946	0.4925	3.6523	4.8304	3.1172	3.5001
Tertiary education	1.0491	1.0245	0.7017	0.3958	0.8271	0.6336	0.2656	0.2599	7.3091	16.0224	1.2380	2.0591
Employed	1.0750	0.4998	0.8921	0.2709	0.6371	0.2754	0.6547	0.3148	0.2977	0.3094	0.4226	0.3016
5+ household size	2.7367**	1.3242	1.1987	0.3603	1.3905	0.5499	0.8822	0.4086	3.7812	4.0300	2.5163	1.6182
Index quintile 2	0.5412	0.2913	0.7519	0.2736	0.7406	0.3527	0.6004	0.3349	0.0700*	0.1077	0.5701	0.4458
Index quintile 3	0.4732	0.2712	0.4915*	0.1978	0.5804	0.2981	0.5009	0.3023	0.0387**	0.0617	0.1985	0.2312
Index quintile 4	1	–	0.3054**	0.1517	0.3211*	0.2153	0.3737	0.2862	1	–	0.7353	0.6452
Index quintile 5	0.4553	0.4103	0.2989**	0.1672	0.3558	0.2657	0.8386	0.6068	0.2136	0.4225	0.7142	0.9356
Sample (n)	256		297		297		295		198		297	
Pseudo R^2^	0.145		0.0574		0.0596		0.0743		0.4151		0.1758	

Notes: Results are for logistic regression models. ***p < 0.01; **p < 0.05; *p < 0.1, OR - Odds ratio, SE - Standard error. Approach 1 estimates catastrophic health expenditure using health costs only, while approach 2 uses health costs plus transport costs. Ataguba method (y = 0.8) - threshold varies with household expenditure. Ataguba method (y = 1) - constant threshold of 10%

Using the WHO method, for approach 1 we see that the factors that increase the odds of catastrophic health expenditure among diabetic patients are being female (Odds ratio [OR] 3.72; Standard error [SE] 2.06), not having children (OR = 4.01; SE = 2.71) and a household size of five or more people (OR = 2.74; SE = 1.32). Patients who were non-African had reduced odds of experiencing catastrophic health expenditure due to diabetes care (OR = 0.16; SE = 0.30). Applying the WHO method to approach 2, we find that female diabetic patient had increased odds of experiencing catastrophic health expenditure (OR = 1.73; SE = 0.52). Falling within the third wealth quintile (OR = 0.49; SE = 0.20), fourth wealth quintile (OR = 0.31; SE = 0.15) and fifth wealth quintile (OR = 0.30; SE = 0.17) were associated with reduced odds of experiencing catastrophic health expenditure. In both the regressions based on the estimates of catastrophic health expenditure calculated with Ataguba’s method, diabetic patients who reported not having any children had increased odds of experiencing catastrophic health expenditure for diabetes care (OR = 3.36; SE = 1.83 when ƴ = 0.8 and OR = 3.54; SE = 2.17 when ƴ = 1).

Due to the low incidence of impoverishment when calculated using the WHO method and the loss of statistical power, we present results for the multivariate logistic regressions of impoverishment calculated using the Ataguba method. Using approach 1 (excluding transport costs) patients who were female (OR = 12.83; SE = 15.94) and had no children (OR = 20.38; SE = 34.50) had increased odds of experiencing impoverishment due to diabetes care. Patients who were single (OR = 0.01; SE = 0.01), within the second (OR = 0.07; SE = 0.11) and third (OR = 0.04; SE = 0.06) wealth quintiles had reduced odds of experiencing impoverishment due to diabetes healthcare costs. Using approach 2 (health costs plus transport costs), diabetic patients who reported being single (OR = 0.30; SE = 0.19) had lower odds of impoverishment due to diabetes healthcare.

## Discussion

This study illustrates the burden of diabetes healthcare amongst patients attending diabetes clinics at subsidised tertiary public healthcare hospitals in Tshwane, South Africa. To the best of our knowledge it is the first study that assesses the incidence of catastrophic health expenditure amongst diabetic patients and assesses inequalities in catastrophic health expenditure. Our study shows that although considerable progress has been made in health service delivery through for example the provision of free healthcare when accessing primary healthcare [23], failures still exist in making healthcare affordable for chronic patients within public hospitals. Our findings show that despite the applied Uniform Payment Fee Schedule (UPFS) in public sector hospitals, there is a high incidence of catastrophic healthcare expenditure amongst diabetic patients. To the best of our knowledge there are no previous studies that estimated the incidence of catastrophic healthcare expenditure amongst diabetic patients in South African public hospitals, we are therefore not able to determine if these inequalities are changing.

We find that the existing UPFS has been ineffective in protecting diabetic patients in low socio-economic groups from financial hardship resulting from diabetic healthcare costs. This finding is consistent with a study that examined health expenditure patterns in rural South Africa and also found high healthcare costs burdens within poorer wealth quintiles [13]. This is of concern given that diabetic patients make multiple visits to healthcare centres [50] and have to contend with diabetes related healthcare costs throughout their life. Although we only focus on indirect costs due to time lost seeking care and time taken off work due to diabetes our finding that indirect costs of diabetes are much higher than the direct costs is consistent with other studies that used national diabetes prevalence estimates to measure indirect costs due to disability, premature mortality and loss of income [17]. Our results also show that transport costs contribute over 50% of the direct diabetes healthcare costs, which results in much greater levels of catastrophic health expenditure and impoverishment when these costs are considered healthcare costs. Transport costs have also been found to be high in other healthcare studies in South Africa [6, 7, 13]. For example, using data from the Agincourt Health and Demographic Surveillance site in South Africa, Goudge et al. finds that transport costs contribute 42% to health expenditure [13]. These high costs point to the urgent need for interventionist programmes to improve access to healthcare for chronic patients who make multiple visits to healthcare centres via provision of free or subsidised patient transport services.

Consistent with other studies we find that the incidence of catastrophic health expenditure is sensitive to the method used [29, 42, 49]. Given the arbitrary nature of threshold selection, the high poverty levels amongst Africans in Tshwane [26], the high transport costs and the high reliance on social grants (30.3% of households) [51], our discussion focuses on the catastrophic health expenditure measured using two methods: (1) healthcare costs plus transport costs at a 10% threshold of capacity to pay; (2) healthcare costs plus transport costs at a variable threshold of total household expenditure. We argue that in this setting, expenditure as low as 10% of capacity to pay or any expenditure at all is catastrophic.

The incidence of catastrophic health expenditure due to diabetes has previously been investigated in other countries [52, 53]. Using the WHO method and a threshold of 10%, the incidence of catastrophic health expenditure due to diabetes care was 25%. On the other hand, the Ataguba method yields a lower incidence of catastrophic health expenditure due to diabetes of 13%. This finding is similar to other studies that find that the incidence of catastrophic health expenditure is higher when measured as a proportion of capacity to pay (non-food expenditure) compared to when measured as a proportion of total household expenditure [42, 54, 55]. The capacity to pay method displays a greater ethical concern for the poor by recognising the higher spending on essential items by poor households when compared to richer households [5, 42].

Consistent with results from a systematic review by Njagi et al., an assessment of the patterns and socioeconomic inequalities in catastrophic health expenditure and impoverishment due to diabetes in our study, showed that the incidence is highest within the lowest socio-economic groups [49]. Using a cross sectional sample of 308 type 2 diabetic patients attending a tertiary healthcare institution in Nigeria, Okoronkwo et al. also find that the incidence of catastrophic health expenditure is highest within the lowest socio-economic group [18]. Although we focused on diabetes only, our finding is similar to other studies that find negative CIs for catastrophic health expenditure [56–58] and impoverishment [57, 58]. This means that catastrophic expenditure due to diabetes healthcare is concentrated amongst the poor as indicated by the negative CI and odds ratios for wealth. To the best of our knowledge there is no study that has investigated inequalities in catastrophic health expenditure due to diabetes in South African public hospitals. We are thus unable to make comparisons with earlier inequality estimates. The results from our study also showed that depending on the method used, up to 4% experience impoverishment due to diabetes healthcare. This finding is comparable to other African studies that assessed the impoverishing effects of healthcare using national datasets [40, 41, 59]. This means that up to 4% of households are negatively affected and forced into poverty as a result of diabetes healthcare costs. This is a huge problem particularly in households that do not only incur diabetes related costs but other health related costs.

Our study also provides insight into the determinants of catastrophic health expenditure and impoverishment amongst diabetics visiting public tertiary healthcare facilities. When measuring catastrophic health expenditure as healthcare costs plus transport costs at a 10% threshold of capacity to pay and healthcare costs plus transport costs at a variable threshold of total household expenditure we found that gender, wealth and children were the variables associated with catastrophic health expenditure. Our finding that households within the higher wealth quintile have reduced odds of incurring catastrophic expenditure, is intuitive and consistent with a study by Babikir et al. that makes use of the National Income Dynamics Survey [12]. Patients who reported not having any children, had increased odds of incurring catastrophic health expenditure and impoverishment. Although the study did not distinguish between young and old or employed and unemployed children, our finding may point to the role that family or social networks play in assisting with healthcare costs. We found that being female was associated with an increased odds of catastrophic health expenditure and that being single was associated with a reduced odds of impoverishment. We find that when compared to the first quintile the odds of being impoverished for those in the fourth and fifth quintile is statistically insignificant. This finding is not consistent with previous literature [60] and calls for further investigations.

Our study has some limitations. Due to the recall periods, results may be subject to recall bias. Our study focused on those who used healthcare services, thus, excluding those with undiagnosed diabetes who still incur healthcare costs as a result of other illnesses that could be related to diabetes. Our study does not take into account other healthcare costs that may be incurred by patients and are not related to diabetes. The exclusion of observations with missing data in variables used for the construction of catastrophic health expenditure or impoverishment may have introduced bias in our study results. Also, our study is not nationally representative as it does not reflect the costs associated with diabetes care in private outpatient clinics. The resulting impact is that the findings from this study cannot be generalised as they are not applicable to diabetic patients who access private healthcare facilities where medical insurance is a common healthcare payment method. Furthermore as reflected by the recorded response rate of 81%, the risk of selection bias cannot be ignored. A comparison of our final analytic sample and refusals showed that our final analytic sample contained more Africans (76% versus 72%) and contained less females (61% versus 62%). Socio-demographic differences between the final sample and refusals could have introduced bias in the study. The methods used in our study have been criticised for ignoring the role of savings, assets, family and friends in healthcare financing [42, 61]. Although recent studies encourage the use of a range of equivalence scales when using the WHO method our study only applies one scale. Despite these shortcomings, these methods provide useful measures of catastrophic health expenditure [42] that inform policy makers on the need for financial risk protection amongst diabetics visiting public hospitals. Our study may form the basis for future investigations across the public healthcare facilities in South Africa. Further qualitative research is needed to complement the quantitative findings from this study, in particular to capture the complex nature of factors associated with patients’ diabetes management.

## Conclusion

Our results are an important contribution to the literature on diabetes costs amongst patients visiting public hospitals in South Africa. We find that transport costs contribute a significant portion to direct healthcare costs. These high costs point to the urgent need for interventionist programmes to improve access to healthcare for chronic patients who make multiple visits to healthcare centres, such as through subsidised or free transport services. Our study shows that some diabetic patients do incur catastrophic healthcare expenditure and that inequalities in catastrophic health expenditure favour those within more affluent socio-economic groups. We find that financial protection of diabetes patients visiting South African public hospitals is limited due to the difficulty in identifying unemployed individuals to be exempted from paying for health services. These expenditures are quite high considering the high unemployment rates and multiple annual hospital visits by many of the diabetics. Our study also shows that being female and not having children significantly increases the odds of catastrophic health expenditure for diabetes care. Being more affluent reduced the odds of catastrophic health expenditure. These observations suggest health financing interventions amongst diabetic patients should further target the poor and poor women in particular. Thus, a recommendation of the study to government is for the elimination of hospital fees for diabetic patients, which must be accompanied by targeted interventions to reduce transport costs associated with accessing hospitals.

## Additional files

Additional file 1: Outline of variables used in regression analysis. (DOCX 12 kb)

Additional file 2: Catastrophic health expenditure and impoverishment related to diabetes care, by wealth quintile. (DOCX 31 kb)

## Abbreviations

CI: Concentration Index
CTP: Capacity to Pay
FEH: Food Expenditure
HSRC: Human Sciences Research Council
ID: International dollars
NHI: National Health Insurance
OOP: Out of Pocket
SDG: Sustainable Development Goals
THE: Total Household Consumption Expenditure
UPFS: Uniform Payment Fee Schedule
WHO: World Health Organisation
