# Supplementary material for: Incidence, socio-economic inequalities and determinants of catastrophic health expenditure and impoverishment for diabetes care in South Africa: a study at two public hospitals in Tshwane
Source: Int J Equity Health. 2019 May 22;18:73. doi: 10.1186/s12939-019-0977-3 (PMC6530010; doi:10.1186/s12939-019-0977-3)
Supplement: Supplementary file 1 — Outline of variables used in regression analysis. (DOCX 12 kb) [file 12939_2019_977_MOESM1_ESM.docx]

**Additional file 1: Outline of variable used in regression analysis**

| **Independent variable** | **Description** | **Measurement** |
| --- | --- | --- |
| Age | Age of respondent | Continuous variable |
| Gender | Gender of the respondent | Dummy variable: 0=male, 1=female |
| Race | Race of the respondent | Dummy variable: 0=African, 1=non-African |
| Marital status | Marital status of the respondent | Dummy variable: 0=married, 1=single |
| Children | Respondent has children | Dummy variable: 0=yes, 1=no |
| Education | Education level of respondent | Categorical variable: 0=Primary, 1= Secondary, 2=Tertiary |
| Employment status | Employment status of respondent | Dummy variable: 0=unemployed, 1=employed |
| Household size | Total household size of respondent | Dummy variable: 0=1-4 individuals, 1=5+ individuals |
| Index quintile | Wealth index generated via MCA and later categorised into quintiles | Categorical variable: 1=quintile 1, 2=quintile 2, 3=quintile 3, 4=quintile 4, 5=quintile 5 |
