# Supplementary material for: Incidence, socio-economic inequalities and determinants of catastrophic health expenditure and impoverishment for diabetes care in South Africa: a study at two public hospitals in Tshwane
Source: Int J Equity Health. 2019 May 22;18:73. doi: 10.1186/s12939-019-0977-3 (PMC6530010; doi:10.1186/s12939-019-0977-3)
Supplement: Supplementary file 2 — Catastrophic health expenditure and impoverishment related to diabetes care, by wealth quintile. (DOCX 31 kb) [file 12939_2019_977_MOESM2_ESM.docx]

**Additional file 2: Catastrophic health expenditure and impoverishment related to diabetes care, by wealth quintile**

|  | **Approach 1** | | | | | | | **Approach 2** | | | | | | |
| --- | --- | --- | --- | --- | --- | --- | --- | --- | --- | --- | --- | --- | --- | --- |
| **Indicator** | **Wealth quintiles** | | | | |  |  | **Wealth quintiles** | | | | |  |  |
|  | **1** | **2** | **3** | **4** | **5** | **Total** | **P-value** | **1** | **2** | **3** | **4** | **5** | **Total** | **P-value** |
| **WHO Standard method** |  |  |  |  |  |  |  |  |  |  |  |  |  |  |
| Poor | 13.33% | 5.95% | 7.14% | 1.30% | 0.00% | 5.87% | 0.0000 | 13.33% | 5.95% | 7.14% | 1.30% | 0.00% | 5.87% | 0.0000 |
| Catastrophic 10 % | 18.67% | 9.52% | 7.50% | 0.00% | 5.77% | 8.64% | 0.0000 | 39.73% | 30.12% | 22.97% | 14.06% | 14.29% | 25.36% | 0.0210 |
| Catastrophic 20 % | 9.33% | 5.95% | 2.50% | 0.00% | 3.85% | 4.46% | 0.0000 | 17.81% | 13.25% | 10.81% | 6.25% | 4.08% | 11.08% | 0.0000 |
| Catastrophic 30 % | 4.00% | 4.76% | 2.50% | 0.00% | 3.85% | 3.06% | 0.0500 | 15.07% | 8.43% | 6.76% | 1.56% | 4.08% | 7.58% | 0.0000 |
| Catastrophic 40 % | 4.00% | 2.38% | 1.25% | 0.00% | 3.85% | 2.23% | 0.0000 | 12.33% | 7.23% | 5.41% | 1.56% | 4.08% | 6.41% | 0.0000 |
| Impoverished | 0.00% | 0.00% | 0.00% | 0.00% | 0.00% | 0.00% | - | 4.76% | 2.63% | 0.00% | 3.13% | 0.00% | 2.19% | 0.0510 |
| **Ataguba method (ƴ=0.8)** |  |  |  |  |  |  |  |  |  |  |  |  |  |  |
| Catastrophic head count ratio | 9.21% | 2.35% | 5.00% | 0.00% | 3.85% | 4.14% | 0.0000 | 20.27% | 14.29% | 12.16% | 9.23% | 8.16% | 13.29% | 0.0210 |
| Prepayment poverty head count | 32.88% | 24.69% | 20.27% | 16.92% | 2.08% | 20.82% | 0.0000 | 32.88% | 24.69% | 20.27% | 16.92% | 2.08% | 20.82% | 0.0000 |
| Post-payment poverty head count | 39.73% | 25.93% | 21.62% | 16.92% | 4.17% | 23.17% | 0.0000 | 42.47% | 28.40% | 21.62% | 21.54% | 4.17% | 25.22% | 0.0000 |
| Impoverished | 6.85% | 1.23% | 1.35% | 0.00% | 2.08% | 2.35% | - | 9.59% | 3.70% | 1.35% | 4.62% | 2.08% | 4.40% | - |
| **Ataguba method (ƴ=1)** |  |  |  |  |  |  |  |  |  |  |  |  |  |  |
| Catastrophic head count ratio | 6.67% | 0.00% | 2.50% | 0.00% | 3.85% | 2.49% | 0.0000 | 16.44% | 8.43% | 8.11% | 6.15% | 10.20% | 9.88% | 0.0050 |
| Prepayment poverty head count | 32.88% | 24.69% | 20.27% | 16.92% | 2.08% | 20.82% | 0.0000 | 32.88% | 24.69% | 20.27% | 16.92% | 2.08% | 20.82% | 0.0000 |
| Post-payment poverty head count | 39.73% | 25.93% | 21.62% | 16.92% | 4.17% | 23.17% | 0.0000 | 42.47% | 28.40% | 21.62% | 21.54% | 4.17% | 25.22% | 0.0000 |
| Impoverished | 6.85% | 1.23% | 1.35% | 0.00% | 2.08% | 2.35% | - | 9.59% | 3.70% | 1.35% | 4.62% | 2.08% | 4.40% | - |

Note: Approach 1 estimates catastrophic health expenditure using health costs only, approach 2 uses health costs plus transport costs. Ataguba method (y=0.8) - threshold varies with household expenditure. Ataguba method (y=1) - constant threshold of 10%.
